# Full Tibialis Anterior Muscle Turnover Flap for Distal Tibial Coverage in a High-Risk Patient With Ankle Arthrodesis: A Case Report

**DOI:** 10.7759/cureus.115341

**Published:** 2026-08-28

**Authors:** Danny Kazzazi, Francis Banhindy, Fawz Kazzazi, Samim Ghorbanian, Parvis Sadigh

**Affiliations:** 1 Plastic Surgery, Royal London Hospital, London, GBR

**Keywords:** ankle arthrodesis, distal tibia, limb-salvage surgery, local flap reconstruction, lower-limb reconstruction, soft-tissue coverage, tibialis anterior flap

## Abstract

Distal tibial wounds with exposed bone or hardware pose a reconstructive challenge, particularly in medically high-risk patients unsuitable for free flap reconstruction. The tibialis anterior muscle is rarely used for reconstruction because sacrifice of the muscle may result in loss of ankle dorsiflexion. However, in selected patients with ankle arthrodesis, complete tibialis anterior sacrifice may be more acceptable because active ankle dorsiflexion is no longer essential.

This case report describes a 66-year-old woman with significant cardiopulmonary comorbidity who developed anterior distal tibial wound dehiscence with exposed bone and hardware following revision ankle arthrodesis. After debridement, removal of infected hardware, antibiotic therapy, and negative-pressure wound therapy, definitive coverage was achieved using a full tibialis anterior muscle turnover flap with split-thickness skin grafting. The muscle was mobilised and turned medially over the distal tibial defect and inset without tension. The flap and graft healed successfully, infection was controlled, and the limb was salvaged while the arthrodesis was protected in immobilisation.

In this high-risk patient with an ankle fusion, a full tibialis anterior turnover flap provided reliable coverage of a distal tibial wound without producing a clinically meaningful functional deficit. This case demonstrates how altered biomechanics following ankle arthrodesis may expand the role of local flap reconstruction and offers a useful limb-salvage option when microsurgical reconstruction is undesirable or contraindicated.

## Introduction

Soft-tissue reconstruction of the distal third of the leg remains challenging because of limited local tissue availability, exposure of bone or hardware, and vascular compromise caused by trauma, infection, scarring, or previous surgery [1]. Free tissue transfer is frequently used for complex distal tibial defects, but prolonged microsurgical reconstruction may be unsuitable in patients with significant cardiopulmonary comorbidity or unsuitable recipient vessels.

The tibialis anterior muscle lies adjacent to many anterior tibial wounds but is rarely used for reconstruction because it is the principal dorsiflexor of the ankle and contributes to inversion; complete sacrifice may therefore cause foot drop. A turnover flap involves mobilising the muscle and rotating it into the adjacent defect; in this case, prior ankle arthrodesis made loss of tibialis anterior function more acceptable. Previous reports have therefore focused predominantly on split or partial tibialis anterior muscle flaps intended to preserve function while providing local soft-tissue coverage [2,3]. Other series have demonstrated that tibialis anterior-based flaps can provide reliable coverage for selected tibial defects, including traumatic wounds and burn-related exposures [4,5]. Use of the entire muscle remains rarely described because of the expected donor-site morbidity associated with complete loss of dorsiflexion.

We report the use of the entire tibialis anterior muscle as a turnover flap with split-thickness skin grafting for coverage of an exposed distal tibial defect following failed revision ankle arthrodesis in a medically high-risk patient. Complete sacrifice of the tibialis anterior muscle is rarely described because of the expected functional morbidity. In this case, free tissue transfer was considered undesirable because of the patient's comorbidity burden and compromised local vascularity, while a distally based hemisoleus flap was considered unlikely to provide adequate reach or bulk. The key feature of this case is that the fused ankle rendered the muscle functionally non-essential, allowing the full muscle to be used for coverage without the usual concern of producing a new foot-drop deficit.

## Case presentation

A 66-year-old woman with end-stage post-traumatic ankle arthritis had previously undergone minimally invasive left ankle arthroscopic arthrodesis. This failed to unite and was followed by open revision ankle fusion with anterior plate fixation. Her comorbidities included ischaemic heart disease with previous myocardial infarction, hypertension, chronic obstructive pulmonary disease, and previous pulmonary embolism requiring long-term anticoagulation.

Approximately one month postoperatively, the anterior ankle wound dehisced with exposure of the distal tibia and hardware. Cultures grew *Staphylococcus aureus*. Despite antibiotic therapy and local wound care, the defect persisted. The patient was managed jointly by orthopaedic and plastic surgery teams. The ankle fusion plate and screws were removed during debridement because of concern for deep infection and nonunion. Antibiotic-impregnated beads were inserted into the fusion site, and a negative-pressure wound dressing was applied.

Following serial debridement and interval wound optimisation, the defect persisted with exposed distal tibia and a deep anterior cavity, requiring definitive vascularised soft-tissue coverage. Handheld Doppler assessment confirmed distal anterior tibial arterial flow, although vascularity appeared compromised by previous surgery and scarring. Given the patient's comorbidity burden, infection, and compromised local vascularity, free flap reconstruction was considered high risk. A distally based hemisoleus flap was considered but was not felt to provide reliable reach or sufficient volume for the anterior ankle defect. The tibialis anterior muscle remained intact and immediately adjacent to the wound. Because the ankle had been arthrodesed, active ankle dorsiflexion was no longer required. A full tibialis anterior muscle turnover flap was therefore selected for definitive coverage.

Written informed consent was obtained from the patient for the publication of this case report and accompanying images. Institutional review board approval was not required for this single-patient case report.

Operative technique

The procedure was performed under general anaesthesia with a thigh tourniquet used during muscle dissection/flap elevation. The existing anterior wound was extended as required, and the distal tibia and fusion site were exposed and thoroughly debrided. The tibialis anterior muscle was identified along the anterolateral tibia. A longitudinal incision was made along the lateral border of the muscle, and the muscle was released from its lateral intermuscular attachments while preserving the remaining medial segmental musculovascular attachments. The distal tendon insertion was partially released to gain additional reach, while the proximal origin remained attached to the upper tibia. This created a medially based tibialis anterior turnover flap pedicled on the preserved medial musculovascular attachments.

**Figure 1:**
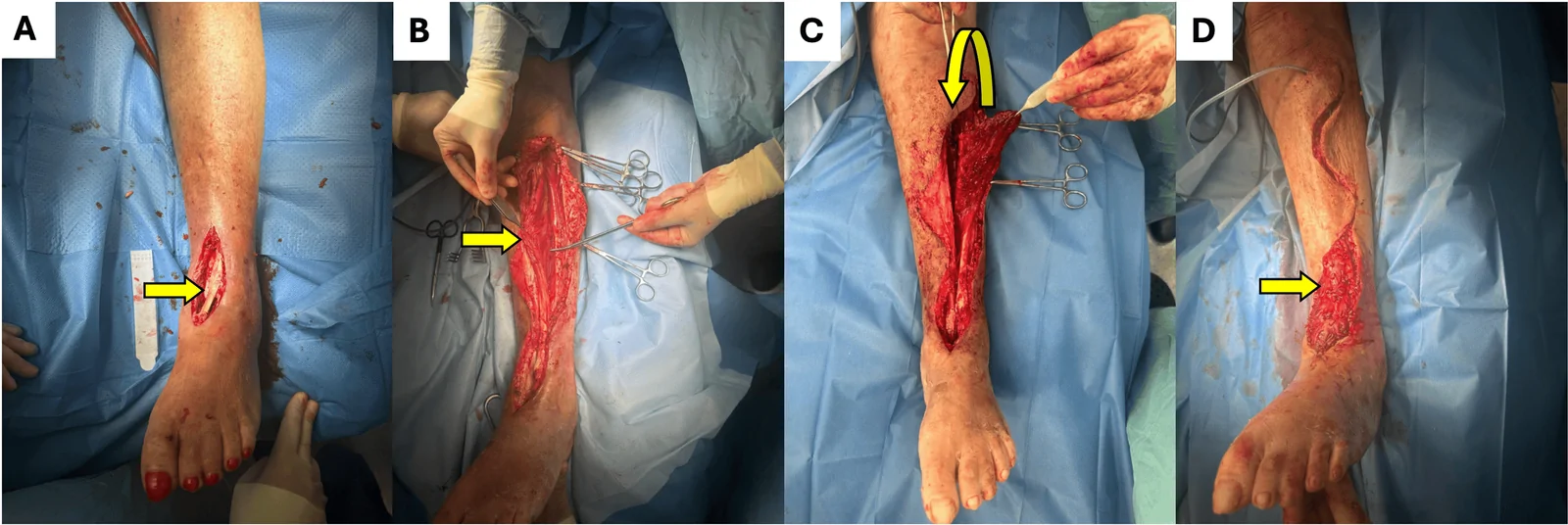
Intraoperative photographs demonstrating stages of full tibialis anterior muscle turnover flap reconstruction (left leg) (A) Anterior distal tibial wound with exposed bone following debridement; arrow indicates the exposed distal tibia. (B) Elevation of the tibialis anterior muscle from the lateral aspect of the tibia; arrow indicates the tibialis anterior muscle. (C) Medial turnover of the muscle flap to provide vascularised coverage of the tibial defect; curved arrow indicates the direction of flap turnover. (D) Final inset of the flap prior to split-thickness skin grafting; arrow indicates the final flap inset.

The muscle was then turned medially and draped over the anterior distal tibia and ankle defect, filling the dead space and covering the exposed bone. Following tourniquet release, the muscle demonstrated punctate bleeding and satisfactory colour. The flap was secured to surrounding fascia and periosteum using absorbable sutures. A meshed split-thickness skin graft harvested from the thigh was applied over the muscle and secured. A negative-pressure bolster dressing was placed over the graft to immobilise the reconstruction and minimise seroma or haematoma formation. The intraoperative stages of defect preparation, tibialis anterior muscle elevation, medial turnover, and flap inset are shown in Figure 1.

Postoperative course and outcome

Postoperatively, the limb was immobilised in a splint with the ankle maintained in neutral alignment, and the patient remained non-weight-bearing. Antibiotics were continued according to culture results. Anticoagulation was managed perioperatively, and therapeutic low-molecular-weight heparin was restarted after surgery without postoperative bleeding complications.

At two weeks, approximately 95% skin graft take was observed, with a small superficial distal area managed successfully with dressings. By one month, the wound was closed, and the flap was epithelialised beneath the graft. At approximately eight weeks, the reconstruction remained healed with no evidence of recurrent infection. The limb was protected in a below-knee cast while fusion progressed. At three months, the patient commenced protected weight-bearing in a controlled ankle motion boot. Radiographs obtained at four months demonstrated progressive fusion across the tibiotalar joint. She initially mobilised short distances using a cane and later used a custom rocker-bottom orthosis. At the one-year follow-up, the wound and graft remained fully healed, with no clinical or radiographic evidence of recurrent infection or osteomyelitis, and radiographs confirmed union across the tibiotalar arthrodesis. The patient had returned to her baseline activities of daily living and was mobilising independently without assistance, with no clinically significant additional functional deficit attributable to the sacrifice of the tibialis anterior muscle.

A summary of the clinical timeline is presented in Table 1.

**Table 1:** Clinical timeline

Time point	Event
Initial surgery	Left ankle arthroscopic arthrodesis
Subsequent course	Nonunion following primary arthrodesis
Revision surgery	Open revision ankle arthrodesis with anterior plate fixation
Approximately one month post-revision	Anterior wound dehiscence with exposed distal tibia and hardware; cultures grew *Staphylococcus aureus*
Following debridement	Hardware removal, antibiotic beads, antibiotics, and negative-pressure wound therapy
Definitive reconstruction	Full tibialis anterior muscle turnover flap with split-thickness skin grafting
Two weeks after reconstruction	Approximately 95% skin graft take
One month after reconstruction	Wound closed and epithelialised
Four months after reconstruction	Progressive radiographic tibiotalar fusion
One year after reconstruction	Wound healed, no evidence of recurrent infection or osteomyelitis, arthrodesis united, and independent mobilisation

Patient perspective

The patient reported relief that the wound had healed and that amputation had been avoided. She described the foot as stiff, consistent with the intended arthrodesis, but did not report a new functional deficit attributable to loss of tibialis anterior function. She remained under orthopaedic and plastic surgery follow-up and continued rehabilitation for gait, conditioning, and orthotic support.

## Discussion

This case demonstrates that a full tibialis anterior muscle turnover flap can provide effective distal tibial coverage in carefully selected patients in whom tibialis anterior function is no longer essential. In the present case, significant medical comorbidity, infection, previous surgery, and compromised local vascularity made prolonged microsurgical free tissue transfer undesirable. The flap provided well-vascularised local tissue, filled dead space, and achieved durable coverage of exposed distal tibia after infected hardware removal. Importantly, the usual disadvantage of sacrificing tibialis anterior function was minimised because the ankle had already been fused.

The literature regarding tibialis anterior muscle flaps remains limited but supportive. Lo et al. described split anterior tibial muscle flaps for exposed tibia in leg avulsion injuries, reporting wound healing with preserved muscle function [2]. Hallock later described the sagittal split tibialis anterior flap as a technical refinement intended to preserve function and local vascularity [3].

Ebraheim et al. reported the use of the tibialis anterior as a local muscle flap for exposed tibia after soft-tissue loss, with complete wound coverage in their series [4]. Sood et al. also described successful tibialis anterior muscle flap coverage for full-thickness tibial burns, with only minimal reported ankle functional deficit [5].

More recent reports have emphasised partial or split techniques to expand the role of the muscle while preserving dorsiflexion, including the split tibialis anterior open-book flap and the split hemianterior tibialis turndown flap [6,7].

A summary of previously reported tibialis anterior muscle flap techniques for tibial wound coverage is presented in Table 2.

**Table 2:** Summary of reported tibialis anterior muscle flap techniques for tibial wound coverage

Author (year)	Patient/indication	Flap technique	Outcome
Lo et al. (1993) [2]	Four patients with leg avulsion injuries and exposed tibia	Split tibialis anterior rotation flap; half of the muscle used as a turnover flap and half retained	All wounds healed; function preserved without foot drop
Hallock (2002) [3]	Technical report for mid-tibial defect	Sagittal split tibialis anterior muscle flap	Wound covered; proposed as a function-preserving local alternative
Ebraheim et al. (2003) [4]	Twelve legs in 11 patients with open middle/distal tibial fractures and exposed bone	Anterior tibialis rotational flap for local coverage	Complete wound coverage and healing reported
Sood et al. (2003) [5]	Five legs in four patients with full-thickness tibial burns after failed grafting	Tibialis anterior muscle flap for longitudinal tibial exposure	Stable coverage in all wounds; minimal reported ankle deficit
Panse et al. (2012) [6]	Five patients with narrow middle-third tibial defects after trauma	Split tibialis anterior "open-book" flap	All wounds covered; no flap failures
Gundlapalli et al. (2014) [7]	Single case with distal anterior ankle wound	Split hemianterior tibialis turndown muscle flap	Wound covered with active dorsiflexion preserved
Present case	High-risk patient with exposed distal tibia following infected revision ankle arthrodesis	Full tibialis anterior muscle turnover flap with split-thickness skin grafting; unlike previous reports focused on split or partial techniques, complete muscle turnover was used because the ankle was fused and tibialis anterior function was no longer essential	Wound healed, arthrodesis united, infection controlled, and limb salvaged without clinically apparent additional functional deficit

Compared with other local options, the tibialis anterior flap is particularly attractive for anterior tibial and anterior ankle defects because it is adjacent to the recipient site and provides vascularised muscle capable of filling dead space. The distally based hemisoleus flap remains useful for selected distal-third defects but may not reliably reach anterior ankle wounds or provide sufficient bulk [8]. The peroneus brevis flap may be appropriate for lateral defects but offers less volume [9]. The key limitation of the tibialis anterior flap is donor morbidity: in a patient with an intact ankle, complete sacrifice may cause foot drop and may require bracing. In contrast, this patient's arthrodesis substantially altered the risk-benefit balance. This case also illustrates the importance of patient-specific reconstructive planning. Although free tissue transfer remains valuable for complex distal lower-limb defects, the reconstructive ladder should not be applied rigidly; rather, the most appropriate option should be selected according to patient factors, defect characteristics, and operative risk [10].

Several technical principles are important. First, the flap should be elevated with the preservation of as many segmental vascular attachments as possible. Second, the inset should be tension-free, which may require release of the distal tendon or additional mobilisation. Third, the reconstruction should be protected postoperatively with immobilisation to prevent shear across the grafted muscle and to protect the underlying arthrodesis. In the present case, the fused ankle provided additional stability, although immobilisation was still used.

The main limitation of this report is that it describes a single patient with a highly specific combination of factors: ankle arthrodesis, exposed distal tibia, infection, comorbidity limiting free flap reconstruction, and an adjacent intact tibialis anterior muscle. The technique should therefore not be generalised to all distal tibial defects. In patients with a functional ankle, split-muscle techniques or alternative flaps may be preferable. Longer follow-up would also be valuable to assess gait, orthotic dependence, and fusion durability. Nevertheless, the case illustrates that well-established local reconstructive options may solve a modern reconstructive problem when patient selection is appropriate.

## Conclusions

Full tibialis anterior muscle turnover provided reliable vascularised coverage in this patient and may provide a valuable local reconstructive option for distal tibial defects in carefully selected patients in whom tibialis anterior function is no longer essential. In medically high-risk patients with ankle arthrodesis, this technique may avoid prolonged microsurgical free tissue transfer while achieving durable soft-tissue coverage and limb salvage. This case further highlights how altered limb biomechanics following arthrodesis can expand the role of local flap reconstruction in complex lower-limb defects. Further cases with longer follow-up and formal functional assessment would help define its broader applicability.
